# Conformational Behavior of SARS-Cov-2 Spike Protein Variants: Evolutionary Jumps in Sequence Reverberate in Structural Dynamic Differences

**DOI:** 10.1021/acs.jctc.3c00077

**Published:** 2023-03-16

**Authors:** Alice Triveri, Emanuele Casali, Elena Frasnetti, Filippo Doria, Francesco Frigerio, Fabrizio Cinquini, Silvia Pavoni, Elisabetta Moroni, Filippo Marchetti, Stefano A. Serapian, Giorgio Colombo

**Affiliations:** † Dipartimento di Chimica, Università di Pavia, via Taramelli 12, 27100 Pavia, Italy; ‡ Department of Physical Chemistry, R&D Eni SpA, via Maritano 27, 20097 San Donato Milanese (Mi), Italy; § Upstream & Technical Services—TECS/STES—Eni Spa, via Emilia 1, 20097 San Donato Milanese (Mi), Italy; ∥ SCITEC-CNR, via Mario Bianco 9, 20131 Milano, Italy

## Abstract

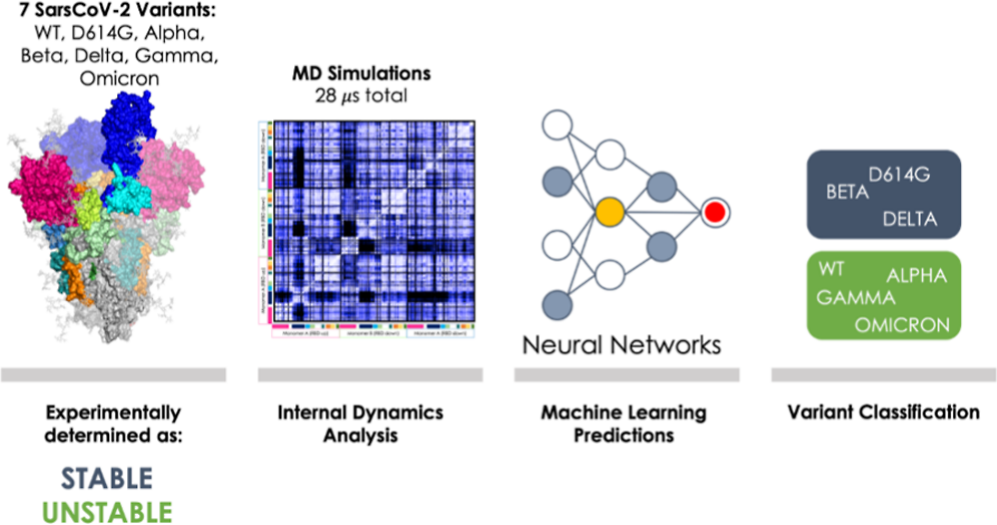

SARS-CoV-2 has evolved rapidly in the first 3 years of pandemic diffusion. The initial evolution of the virus appeared to proceed through big jumps in sequence changes rather than through the stepwise accumulation of point mutations on already established variants. Here, we examine whether this nonlinear mutational process reverberates in variations of the conformational dynamics of the SARS-CoV-2 Spike protein (S-protein), the first point of contact between the virus and the human host. We run extensive microsecond-scale molecular dynamics simulations of seven distinct variants of the protein in their fully glycosylated state and set out to elucidate possible links between the mutational spectrum of the S-protein and the structural dynamics of the respective variant, at global and local levels. The results reveal that mutation-dependent structural and dynamic modulations mostly consist of increased coordinated motions in variants that acquire stability and in an increased internal flexibility in variants that are less stable. Importantly, a limited number of functionally important substructures (the receptor binding domain, in particular) share the same time of movements in all variants, indicating efficient preorganization for functional regions dedicated to host interactions. Our results support a model in which the internal dynamics of the S-proteins from different strains varies in a way that reflects the observed random and non-stepwise jumps in sequence evolution, while conserving the functionally oriented traits of conformational dynamics necessary to support productive interactions with host receptors.

## Introduction

The Covid-19 pandemic has been sweeping the world since the beginning of 2020, causing the death of millions of people, disrupting social activities, and impacting on the global economy.

Covid-19 is caused by the respiratory pathogen SARS-CoV-2, an RNA virus of the sarbecoronavirus family.^1,2^ Since its emergence, incredible progress has been made in understanding the main risk factors for the emergence of Covid-19 and in developing approaches to prevent or treat the disease.^3^ After a little more than 1 year from the characterization of the viral sequence, the first effective vaccines appeared, and mass vaccination significantly reduced the number of deaths or of patients with severe conditions (https://covid19.trackvaccines.org/vaccines/).^4,5^ At the end of 2021/beginning of 2022, the first pharmacological treatments also started to make their appearance on the market.^6,7^

All these efforts, especially in the immunological field, were based on studies of viral sequences characterized at the beginning of the emergency. However, viruses are known to evolve, and SARS-CoV-2 is no exception.^8−14^

Over time, several variants have been identified, each characterized by different levels of infectivity and (in some cases) vaccine escape or resistance.^15−19^ The recent Omicron variant (and its subvariants) for instance is significantly more infectious and more vaccine-resistant than the original Wuhan strain.^20−24^

In order to survive, evolution requires that the virus acquire mutations that help it spread more efficiently and circumvent immunity.^8,11,25^

While it was initially expected that new mutants would descend from existing ones through a stepwise process in which new mutations are implanted on successful sequences, sequencing data showed that the newer and more efficient variants (e.g., Omicron and alike) harbor a notably large number of mutations.^24,26^ This represents a key peculiarity of SARS-CoV-2: as noted by Bloom and colleagues (The New York Times “We Study Virus Evolution. Here’s Where We Think the Coronavirus Is Going.” March 28, 2022. https://www.nytimes.com/interactive/2022/03/28/opinion/coronavirus-mutation-future.html), the virus seemed to defy common knowledge with its variants emerging through big evolutionary jumps, at least in the initial steps of diffusion. In this context, it is important to note that there is a great sequence difference between one of the earlier most infective variants, namely Delta, and the later one, i.e., Omicron.

The salient features of the evolution of viral variants of concern (VOC) can effectively be traced to the evolution of the sequence of the Spike protein (S-protein), the glycosylated outer membrane protein which plays the key role in cell entry by interaction with the human receptor angiotensin-converting enzyme 2 (ACE2)^1,27^ and represents the basis for the design of several effective vaccines.^28−31^

In this context, the history of VOC development can in fact be roughly sketched as follows. In early March 2020, the first point mutation appeared, a single amino acid change caused by an A-to-G nucleotide mutation at position 23,403 in the Wuhan reference strain. This mutation gave rise to the emergence of the dominant “D614G” Spike variant, which rapidly spread from Europe to North America, Oceania, and Asia.^32−35^ After this first one, an increased level of surveillance and sequencing contributed to reveal novel variants. Furthermore, other immune escape mechanisms started to be characterized.^36^

Among the ones that have been brought to attention in the last couple of years, the one labeled 20I/501Y.V1 or B.1.1.7, commonly named the Alpha variant, was initially found in the UK and was associated with an increased risk of infection and death.^37^ In South Africa, variant B.1.351 (known as 20H/501Y.V2, Beta) emerged independently from B.1.1.7 but shared some mutations with it.^38^ Next, the P.1 variant (20J/501Y.V3, Gamma) was first identified in travelers from Brazil and featured 17 unique mutations including three in the receptor binding domain of the S-protein, two shared with B.1.351, E484K and N501Y, the latter also shared with the strain of B.1.1.7, and a different mutation K417T which was K417N in the B1.351, Beta strain.^39^ The B.1.617.2 variant (AY, Delta) was first detected in India in late 2020, where it was responsible for a huge surge in the number of cases, and in June 2021 it became the dominant variant globally.^40^ The SARS-CoV-2 Omicron (B.1.1.529, BA.1) variant was first identified on November 24, 2021, in South Africa and immediately declared the VOC replacing the Delta variant. The Omicron variant has a very large number of mutations, around 30-point mutations in the S-protein alone, combined with deletions and insertions of amino acids.^15^

**Figure 1:**
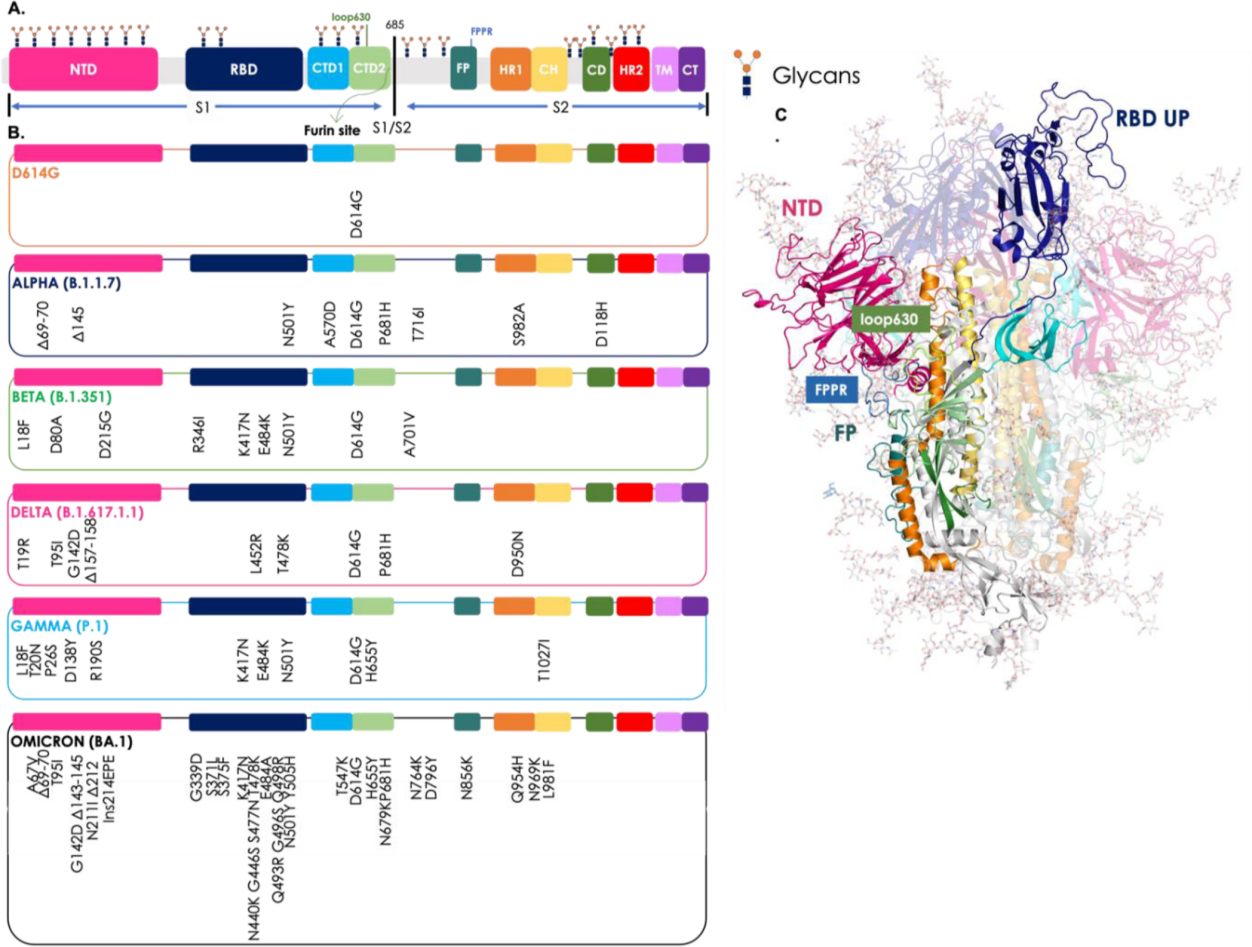
Sequence mutations and structural organization of the S-protein variants studied. (A) Colored-block representation of the sequence of the full-length SARS-CoV-2 S-protein (from PDB ID 6VSB) and its subdivision in the various domains of the S1 and S2 regions: NTD (14–306), RBD (319–528), CTD1 (529–591), CTD2 (592–686), loop 630 (loop630, 620–640), furin cleavage site (S1/S2), FP (788-834), FPPR (828–853), HR1 (910–984), CH (985–1034), and CD (1035–1068). Representative icons (in orange and blue) for glycans in their positions. (B) Positions of all mutations, deletions (Δ) and insertion (ins), from the amino acid sequence of Wuhan in the relative domain of the virus variants studied. The color code of the VOC is D614G in orange, Alpha (B.1.1.7) in blue, Beta (B.1.351) in green, Delta (B.1.617.1.1) in pink, Gamma (P.1) in light blue, and Omicron (BA.1) in black. (C) Full-length, fully glycosylated trimeric structure corresponding to the pdb code 6VSB. Protomer A (RBD “up”): secondary structures are colored by domain as reported above in point (a); protomers B and C (RBD “down”) are transparent. Glycans’ C, N, and O atoms rendered as teal sticks. In the figure, loop630 is shown in light green and FPPR in blue.

The S-protein perturbations associated to the different variants described are summarized Figure 1 (list of the ones studied in this paper). Further variants have emerged and continue to emerge due to the pressure exerted by the virus to adapt and to survive in an increasingly immunized population, such as the Epsilon (B.1.427 and B.1.429), Eta (B.1.525), Iota (B.1.526), Kappa (B.1.617.1), and Mu (B.1.621, B.1.621.1), etc. (https://www.who.int/activities/tracking-SARS-CoV-2-variants).

The differences between the above-mentioned variants have been studied diffusely. Veesler et al. linked the conformational properties to plasma-neutralizing activities.^41^

Recently, a paper by the Amaro and Freeman groups showed that Omicron specifically modified its positive surface charge to improve interactions with heparan sulfates and ACE2.^42^ This effect was related to enhanced binding rates to charged glycocalyx molecules. Other studies have shown that specific mutations in the receptor-binding domain (RBD) can also be correlated to an increased affinity for ACE2.^43−46^

The importance of long-range modulation of S-dynamics was demonstrated to be fundamental in response to the binding of endogenous molecules, such as fatty acids, that were proven to preorganize the RBD for attachment to the receptor.^47−51^

Here, we ask whether the significant sequence differences observed for the various strains reverberate in changes in the traits of long-range structural dynamics of the S-protein by comparing seven different mutant sequences. Specifically, we analyze how the dynamics is modulated by mutations [compared to the initial Wuhan variant, the wild type (WT) in our model] both at the level of global and local motions, specifically focusing on substructures that are important for Spike functions (i.e., recognition of the human receptor ACE2 and conformational reorganization of the architecture to favor host-virus membrane fusion).

To progress along this avenue, we address various aspects of this problem by analyzing and comparing atomistic simulations of S-protein mutants reported in Figure 1. We run four replicas of 1 μs long molecular dynamics (MD) simulations for seven distinct mutants of the S-protein, using the full-length form of the protein with an explicit representation of its glycosylation patterns. The replicas for each mutant were combined, and the respective metatrajectories obtained were used for subsequent analyses. Overall, we sum up a total of 28 μs of explicit solvent MD simulations. Starting from the atomistic resolution investigation of internal fluctuations and analysis of the coordination in the motion of different domains, we demonstrate that different mutants show distinctive dynamic traits, which can be qualitatively correlated to their relative stability properties.

Our results also indicate that the structural dynamics of S-proteins from different strains varies in a way that appears to reflect the jumps in sequence evolution observed at the initial stages of diffusion of the virus.

### Structural Organization of the SARS-CoV-2 S-Protein and Localization of the Mutations

The S-protein is a class I fusion protein, synthesized as a single 1273 amino acid polypeptide chain precursor and subsequently processed by a furin-like protease into the receptor-binding fragment S1 and the fusion fragment S2.^1,2^

Structurally speaking, the SARS-CoV-2 spike associates in a trimer, and it is anchored to the viral membrane by a transmembrane (TM) segment. Furthermore, the protein is heavily glycosylated.^52^

In the prefusion conformation, each monomer is composed of the two subunits S1 and S2 and can be divided into three main topological domains, namely the head, stalk, and cytoplasmic tail (CT) as shown in Figure 1.

At a more refined level of structural detail, S1 folds into four domains, namely the N-terminal domain (NTD), RBD, and two C-terminal domains (CTDs). These wrap around the prefusion S2 structure which includes the fusion peptide (FP), the FP proximal region (FPPR), heptad repeat 1 (HR1), the central helix (CH), the connector domain (CD), heptad repeat 2 (HR2), the TM segment, and the CT.^27^

The RBD is the domain dedicated to making contacts with the human cell receptor ACE2. Structural studies based on cryo-electron microscopy (Cryo-EM) and X-ray crystallography showed two possible conformations for this domain, the “up” and the “down” conformations: the former is a state accessible to the receptor, while the latter represents the receptor-inaccessible state.^1,2,53^ The three NTDs are located at the periphery of the trimer, each making contact with the adjacent RBD.

## Results

### Mutations Modulate the Global Internal Dynamics of the S-Protein Variants

First, we notice that mutations have been shown to impact on S-protein stability. Comparative experimental characterization of SARS-CoV-2 VOCs identifies two sets of variants: stable proteins [shown to elute as a single peak in sodium dodecyl sulfate (SDS)-polyacrylamide gel electrophoresis] which comprise the D614G, Beta, and Delta variants^34,54,55^ and unstable proteins (shown to elute as two or more peaks, some of which correspond to aggregated species due to unfolding/misfolding), which entail the WT, Alpha, Gamma, and Omicron.^55^ Interestingly, Omicron represents one of the most unstable species.^56^

To explore whether dynamic signatures exist that can be related to the observed trends in stability, we set out to characterize residue-pair distance fluctuations (DFs) among all amino acid pairs in various proteins.^57−61^ This calculation, which reports the mean-square fluctuation of the inter-residue distance between any two residues in the protein, informs on the effect of sequence variations on the internal dynamics of the protein. In particular, an increase of global internal flexibility (overall decreased pair coordination) can be related to an enhanced tendency to support transitions to states alternative to the native one. In this framework, sequence alterations reverberate in the differential capacity of the protein to populate the native basin.

Given the complexity of the system under examination and the expectedly wide structural variations involved, our aim is not to sample large conformational changes (or even unfolding pathways and mechanisms) but to provide a simple dynamic-based approximation of global stability. Furthermore, DF analysis can potentially highlight substructures and (ensembles of) residues that respond differently to sequence variations.

We first comparatively analyzed the WT versus the D614G variant.

**Figure 2:**
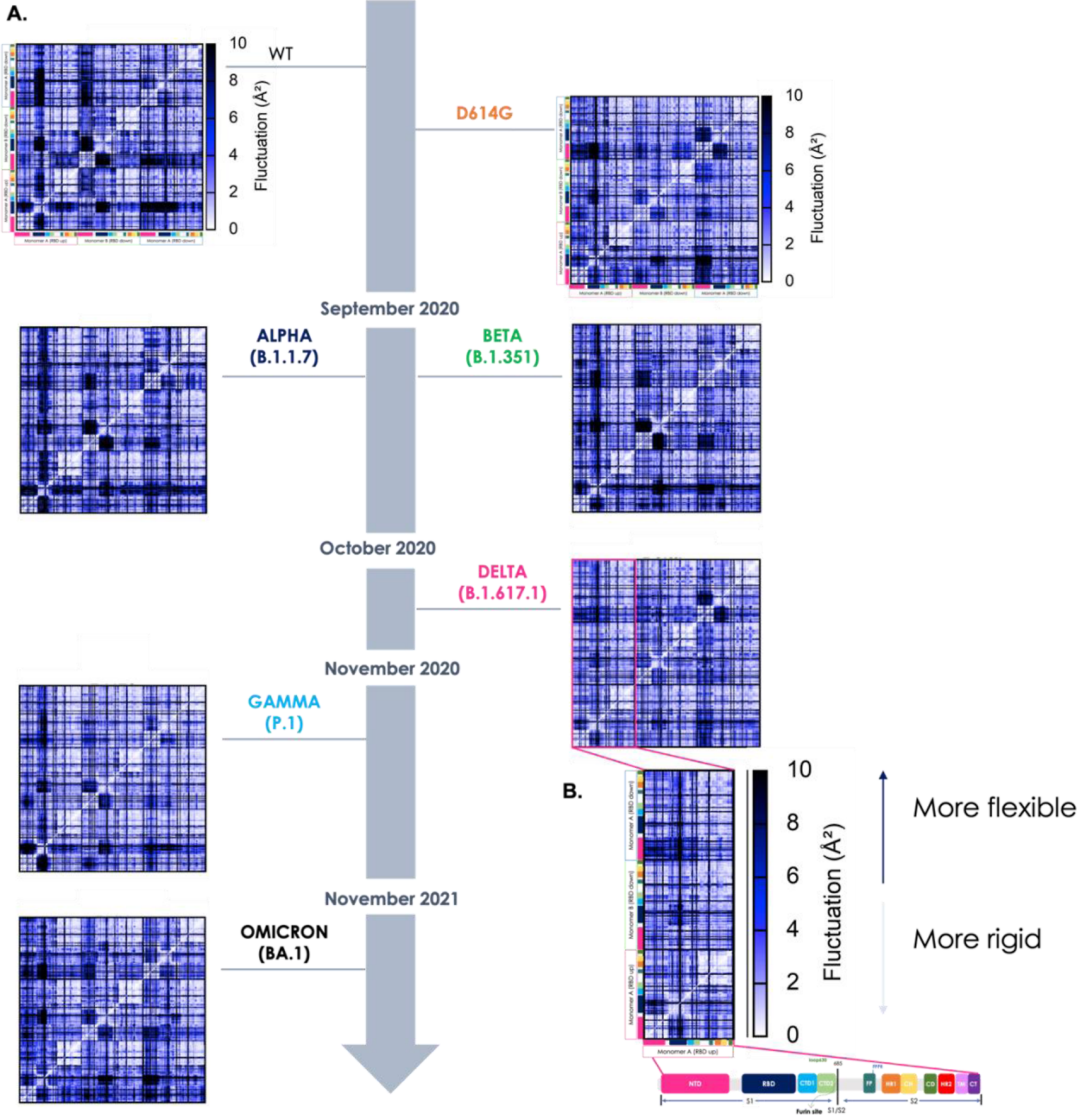
Characterization of the internal dynamics and flexibility of the various Spike mutants in terms of residue-pair fluctuations. (A) Matrix of residue-pair DFs among all amino acid pairs in all variants. The VOCs are represented on the timeline according to the appearance and colored in the color code described in Figure 1. The *x* and *y*-axes show the sequences colored per domain as in Figure 1. Residue pairs with fluctuations between 0 and 2 Å are white, between 2 and 8 Å blue, and larger than 8 Å black. (B) Zoom-in on the submatrix of monomer A (with the RBD up): this shows pair fluctuations within monomer A and with the other two monomers (RBD down), specifically for the Delta variant. The colors (from white to black) represent the intensity of the fluctuation: the clearer the matrix pixel, the more intense the coordination between the amino acid pairs and ultimately more rigid the (region of the) protein; the darker the color, the higher the distance fluctuation indicative of lower coordination. The inset also reports a zoom-in on the color code, reflecting the domain partitioning of single Spike protomers, which also refer to the subdivision of the various axes.

**Figure 3:**
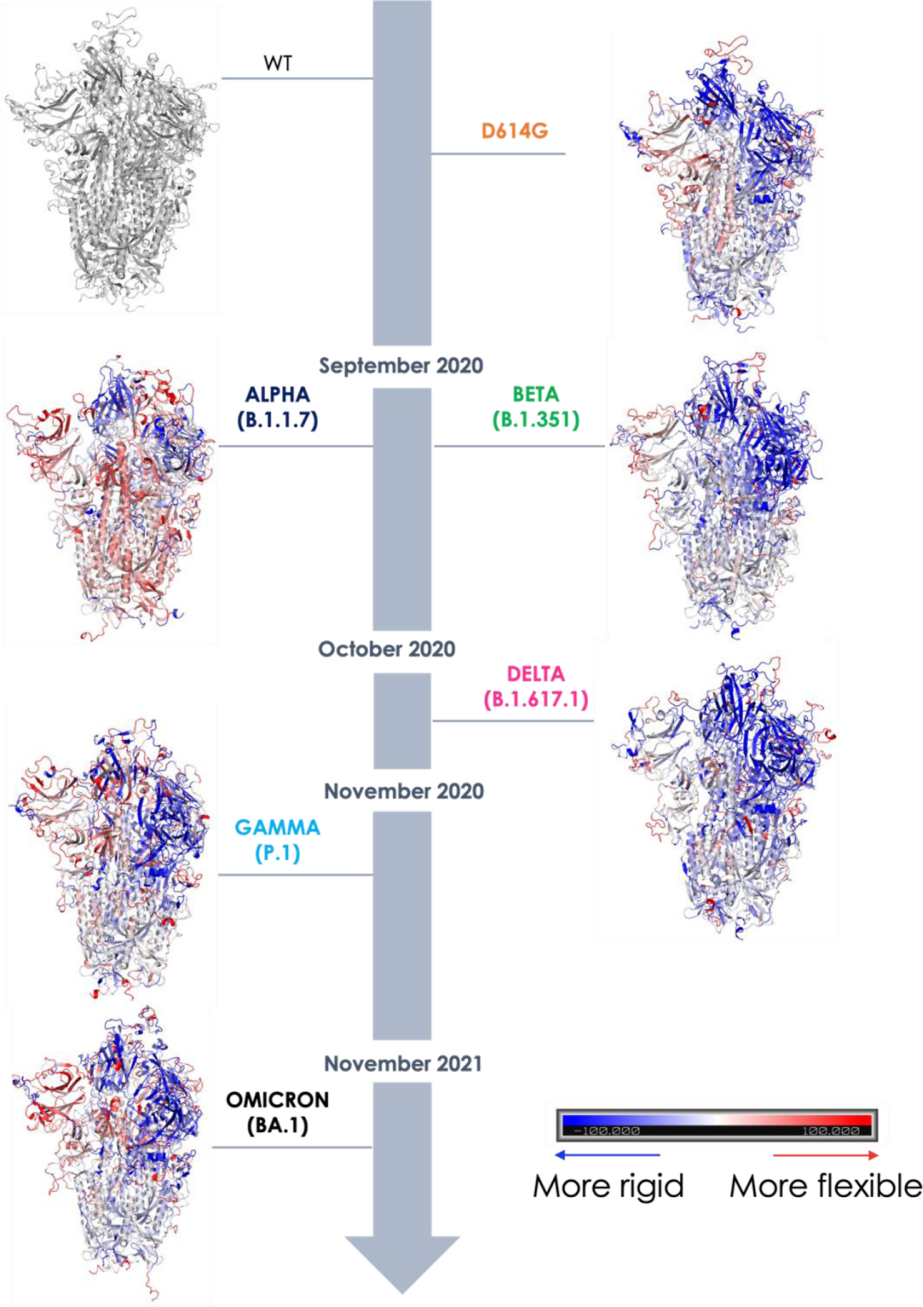
Structural projection of flexibility differences with respect to the WT Wuhan mutant. Point-by-point subtraction of the DF matrix of the WT from the matrix of each variant. The larger the fluctuation value for each residue is for the variant with respect to the WT, the more the domain is colored red. Conversely, the lower the value is in the variant, the more the protein is colored blue: thus blue-colored domains represent areas that are stiffer overall, i.e., where coordination is greater, and this is clearly seen in the more stable variants (D614G, Beta, and Delta). The appearance of red areas reports on the lower coordination and therefore the greater flexibility of the variants (see Alpha, Gamma, and Omicron).

The overall DF matrices show the block character typically observed for multidomain proteins, reflecting the alternation of regions of small and large inter-residue DFs. It is immediately evident that the D614G mutant displays patterns of residue-pair coordination that are significantly more diffuse than in the case of the WT protein (the Wuhan sequence). Indeed, in D614G higher coordination appears to extend to the whole 3D structure of the protein (Figures 2, 3). The pervasive enhancement of pair coordination can contribute to stabilize the protein in the 3D structure of the native state. Breaking-up the extensive networks of low fluctuating residue pairs in D614G can expectedly require a higher energy contribution than in the case of the native sequence.

Extension of the analysis to the Delta variant confirms the trend for more stable proteins to be characterized by more diffuse networks of highly coordinated residue-pairs. The same trends hold for the final stabilized variant studied herein, namely the Beta variant, B.1.351 (Figure 2).

Analysis of the finer details of the matrices can aptly highlight detailed sequence-dependent modulations of the S-proteins. In this context, of particular interest is the finding that in D614G, Beta, and Delta, an increasing coordination with the rest of the protein is observed for the RBD in the “up” conformation, the one required for interaction with human cells.^1^ In this model, mutations induce an overall change in the S-dynamic states that significantly preorganize the protein for recognition of its receptors.

Strikingly, the analysis of DF distributions in Alpha, Gamma, and Omicron, together with WT, shows a trend pointing to increased internal flexibility: larger pair-fluctuations are indeed generally observed. Importantly, the Omicron variant, which experimentally was shown to be one of the least stable and most infective mutants, turned out to be the protein with the larger internal flexibility. Here, the residues of monomer A, in particular, become completely uncoordinated with the rest of the protein.

Interestingly, in all these cases, the RBD in the “up” conformation is seen to maintain similar coordination patterns with the rest of the protein as those observed above for the stabilized mutants.

These data suggest a pattern whereby increased flexibility can be viewed as a double-edged sword. While determining a degree of structural instability, flexibility in general supports the exploration of dynamic states that facilitate conformational conversions. The ability to sample different states eventually increases the probability for displaying the RBDs in the proper orientation for interaction with host-receptor, while at the same time supporting the large conformational rearrangements in the stem region that are required for subsequent membrane fusion.

To provide a more direct structural picture of how global internal dynamics is modulated upon mutation, we set out to calculate the variation in flexibility in the various mutants, using the Wuhan WT protein as a reference. In this context, we carried out a point-by-point subtraction of the WT matrix from the matrix of each mutant. The resulting difference matrix is further manipulated by calculating the sum of all values in each column: as each column corresponds to one residue, the calculation returns a compact description of the increase or decrease of flexibility for each residue in the mutant with respect to the WT. The data are then projected on the structure as reported in Figure 3: a pervasive increase of coordination is clearly observed for Delta, while in contrast, a marked increase of flexibility is noticed for Alpha and Omicron. It is important to underline here that the results of these analyses (and their graphical rendering in Figure 3) are to be considered qualitative: we can only distinguish between more coordinated (stable) structures vs. less coordinated (flexible and unstable) ones without the possibility to build a ranking of the relative stabilities of mutants with respect to one another.

Summarizing, the sequence-dependent modulation of Spike’s internal dynamics, characterized in terms of the degree of coordination in residue pairs, can be related to the tendency for the structure to sample alternative dynamic states while maintaining the RBD preorganized to interact with its host receptors. On the one hand, stabilization of the native structure would be expected to maximize the display of RBD for interaction (a case exemplified by the Delta variant); on the other hand, increased flexibility of the native state, which would aptly lead to destabilization of the structure, could favor the exploration of states that organize the RBD for ACE2 recognition and subsequent structural transitions in the stem region (a case exemplified by the Omicron variant). Finally, our analysis points to the direction of the dynamic behavior of the different variants that appears to follow a (random) step-wise pattern of differentiation similar to that observed for the evolution and selection of mutations.

### Machine Learning Classification of Variant Dynamics–Stability Relationships

The results described above identify distinct internal dynamic profiles of the S-protein as a function of the sequence and define a possible link between the degree of coordination and emerging (in)stability in VOCs. However, these results are still qualitative and rely on an attentive critical investigation of the features of the DF matrices. To put the analysis of dynamics, and the possibility to relate them to specific features, on a more quantitative ground, we set out to develop a machine learning (ML) approach capable of classifying the variants as “STABLE” or “UNSTABLE” simply based on the input of information on internal dynamics. To this end, we resorted to image recognition methods: in this context, the above-reported DF matrices are considered as images to classify. The advantage of using the whole DF matrix as an input image is that it compactly reports on the internal dynamics state of the protein as a whole. It is important to notice that in this framework, small modifications in the sequence that may reverberate in large-scale coordination modifications can potentially be efficiently identified.^57^

**Figure 4:**
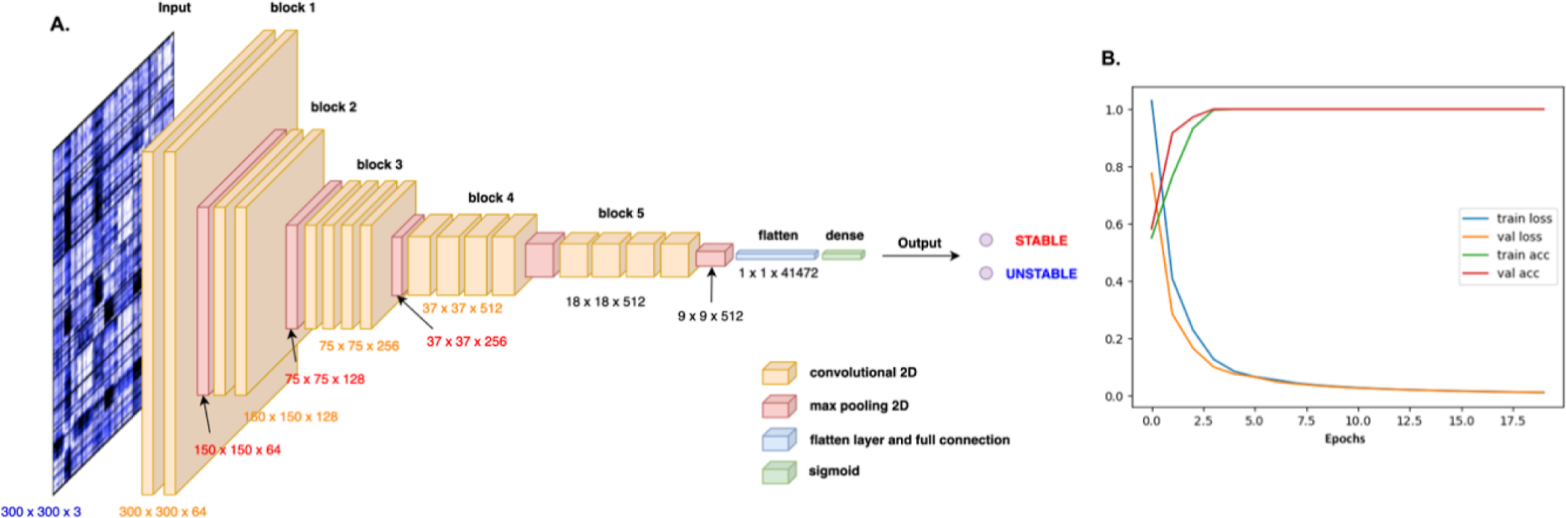
ML approach. (A) Architecture of modified VGG19 model. The pixel image of the DF matrix is transformed in mathematical values which are submitted through convolutional and max pooling layers to reduce the computational cost and overfitting during model training. Flattened fully connected layer is used to converge all data toward the dense output layer, where the sigmoid function acts in order to provide the binary output. (B) Performance evaluation during training and validation of the model. Accuracy achieves values close to 1.0 during both training and validation steps, while in both the cases, the loss function decreases toward 0. The combination of these two pieces of information ensures that the model is well trained and suitable for the next evaluation on the test data set.

We used a convolutional neural networks (CNN) approach. Specifically, we started from the VGG19 model, extensively tested in classification problems and easy to import into in-house Python scripts from the TensorFlow (TF) library.^62^ Moreover, VGG19 shows one of the best compromises between computational cost and accuracy, especially with graphics processing unit (GPU)-compiled TF.^62^ We introduced modifications to the VGG19 model to increase the dimensions of the layers consistent with the pixel number of the input images (see Supporting Information for more details on the number of parameters processed in each single layer). The layout of the model is reported in Figure 4a.

In our approach, the images depicting the DFs were prepared with a typical resolution of 300 × 300 pixels and used as input for the multiple layers of the model where they are processed by alternating convolutions and max-pooling operations, until achieving the last flattened and fully connected layers, which provide the final output.

To train the model, DF images from the last equilibrated 200 ns of each of the four replicas were used as data sets. Variants were first divided into two sets according to the known stability: D614G, Beta, and Delta were initially considered as STABLE; WT, Alpha, Gamma, and Omicron were labeled UNSTABLE. The final goal of the model was in fact to classify a certain protein as STABLE or UNSTABLE, based *only* on the image of one (or more) respective DF matrix.

To prepare the data sets, we extracted a DF image every 10 ns, and considering the number of replicas for each variant, we ended up with a total of 672 images. Starting from this data set, we operated a manual random separation between test (20%), train (64%), and validation (16%) sets (see Materials and Methods for more details).

Our data set was thus composed as follows:

- Training set: 430 DF matrix images (215 STABLE and 215 UNSTABLE)
- Validation set: 108 DF matrix images (54 STABLE and 54 UNSTABLE)
- Test set: 134 DF matrix images (67 STABLE and 67 UNSTABLE)

The performance evaluation of our method on the training and validation sets are shown in Figure 4b. In both cases, we can highlight a mutual fit and convergence between validation and training accuracies as well as losses. By evaluating our trained model with the test set, we got 100% accuracy, taking only 3 s to scan all 134 DF test images. This result was corroborated by the confusion matrix which showed that among 134 total images, 67 test entries were correctly classified as STABLE, while the other 67 as UNSTABLE. We also calculated the Cohen’s kappa coefficient obtaining a κ value equal to 1 (see Figure S4 in the Supporting Information for more details on confusion matrix and Cohen’s kappa expression).

Next, we moved on to feed the model and predictor with new (and completely unseen and unrelated) sets of data. The new set included DF matrices that were calculated on parts of the trajectories that were not used for either the training or the testing reported above. In particular, we selected matrices calculated even on the less equilibrated parts of the trajectories, specifically the ones at the beginning of the production. The new set was thus composed of 20 DF matrix images, of which 10 came from STABLE variants, while another 10 from UNSTABLE variants. Interestingly, the model was able to predict all cases with 100% accuracy in both STABLE and UNSTABLE entries.

The model thus proves to be able to provide a direct labeling of DF matrices, establishing a link between internal dynamics and the property used for classification (stability in this case): from a physical point of view, the model associates a more diffuse and pervasive pattern of internal coordination to the increased stability of the relative protein, speeding up MD analysis and removing human bias in the classification of distinct variants of human proteins.

### Dynamics of RBD and Functional Substructures in Different Mutants

The above-reported analyses indicate that the common trait in the dynamics of all of the different variants entails the preorganized presentation of the RBD. Indeed, such motions underlie binding to the human ACE2 and are thus key for viral entry. Here, we focus on the characterization of the dynamics of the RBDs in the different variants.

**Figure 5:**
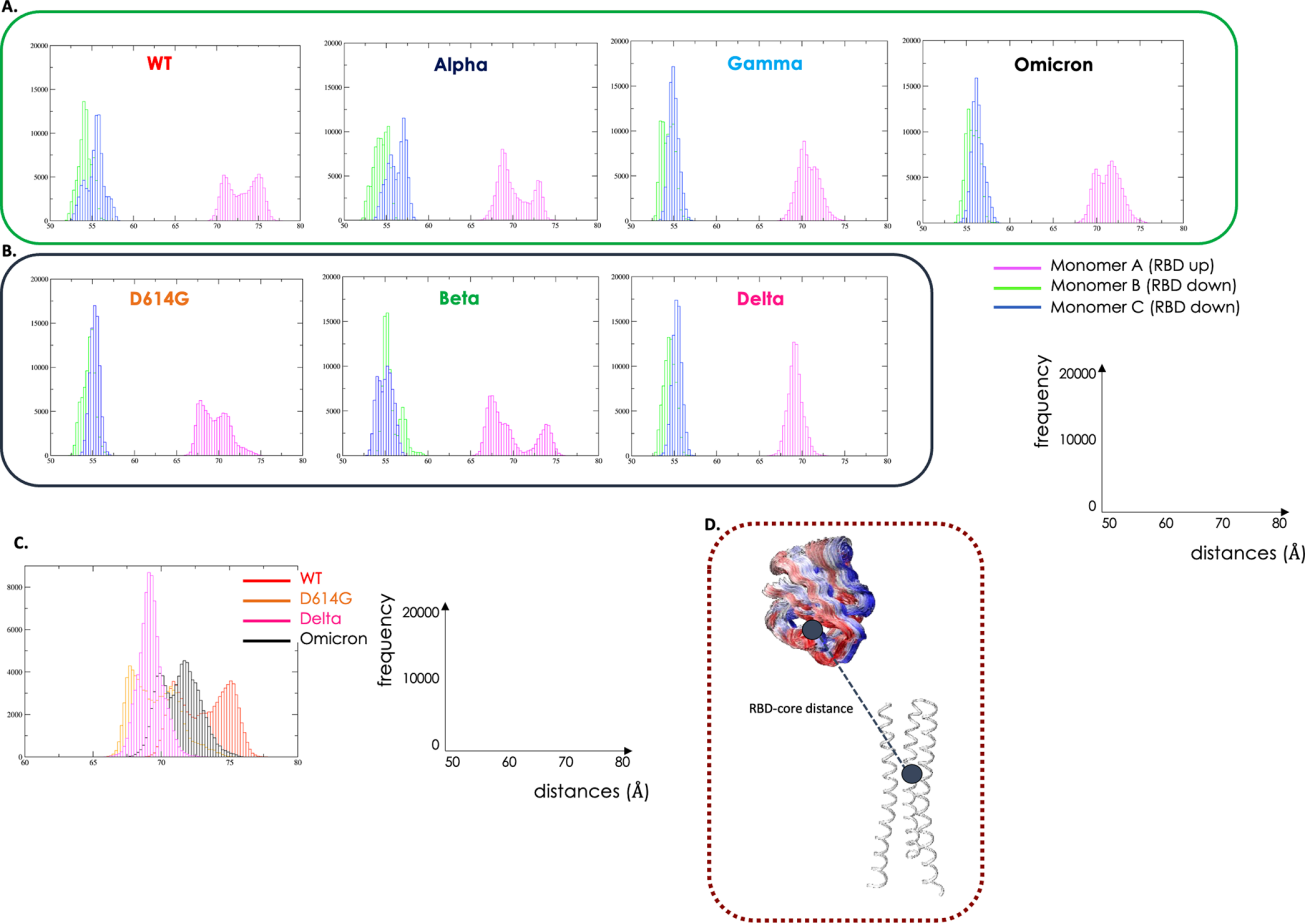
Dynamics of RBD. UNSTABLE variants in green hues and STABLE variants in grey hues. (A) Distance between the COMs of the spike core and of the RBD of the UNSTABLE variants (WT, Alpha, Gamma, and Omicron) represented in histograms. Fluctuation of monomer A with the RBD in the UP position in magenta, monomer B (RBD down) in green, and monomer C (RBD down) in blue. (B) Distance between the COMs of the spike core and of the RBD of the STABLE variants (D614G, Beta, and Delta). The graphs report on the *x*-axis the distances (Å) and on the *y*-axis the frequencies. (C) In this panel, we report the comparison among the distances in the most representative variants (WT, D614G, Delta, and Omicron). (D) Structural representation reporting a simplified cartoon representation of the variable mentioned.

To this end, we monitored the distributions of two variables that recapitulate the main motions of the RBDs with respect to the rest of the protein: the first is the distance between the centers of mass (COMs) of the spike core and of the RBD (see Materials and Methods); the second is the 3D angle between these two parts of the S-protein (see Figure 5 its different subpanels, and Supporting Information).

The COMs distance analysis (Figure 5a,b) shows that there is a tendency for the variant that determines a jump in infectivity, specifically Delta (and to some extent Gamma and Omicron) VOC, which then became dominant on the background of existing variants, to have the RBD in the “up” conformation populating a more restricted part of the conformational landscape.

This tendency can clearly be seen on moving from WT to D614 to the Delta variant (Figure 5b). Strikingly, Delta shows the RBD “up” populating a restricted portion of the available configurations, sticking out of the protein in the direction of possible interaction partners. Albeit to a more limited extent, this is also observed for the Omicron variant. In general, and consistent with the results presented above, the dynamics of the very infectious Omicron variant seem to combine the increase in global conformational flexibility that favors functional conformational transitions with an almost optimal ability to present the RBD for targeting human cell receptors. Both aspects are clearly advantageous for the virus.

Similar trends are also observed for the angle distributions described above (see Supporting Information, Figure S6).

**Figure 6:**
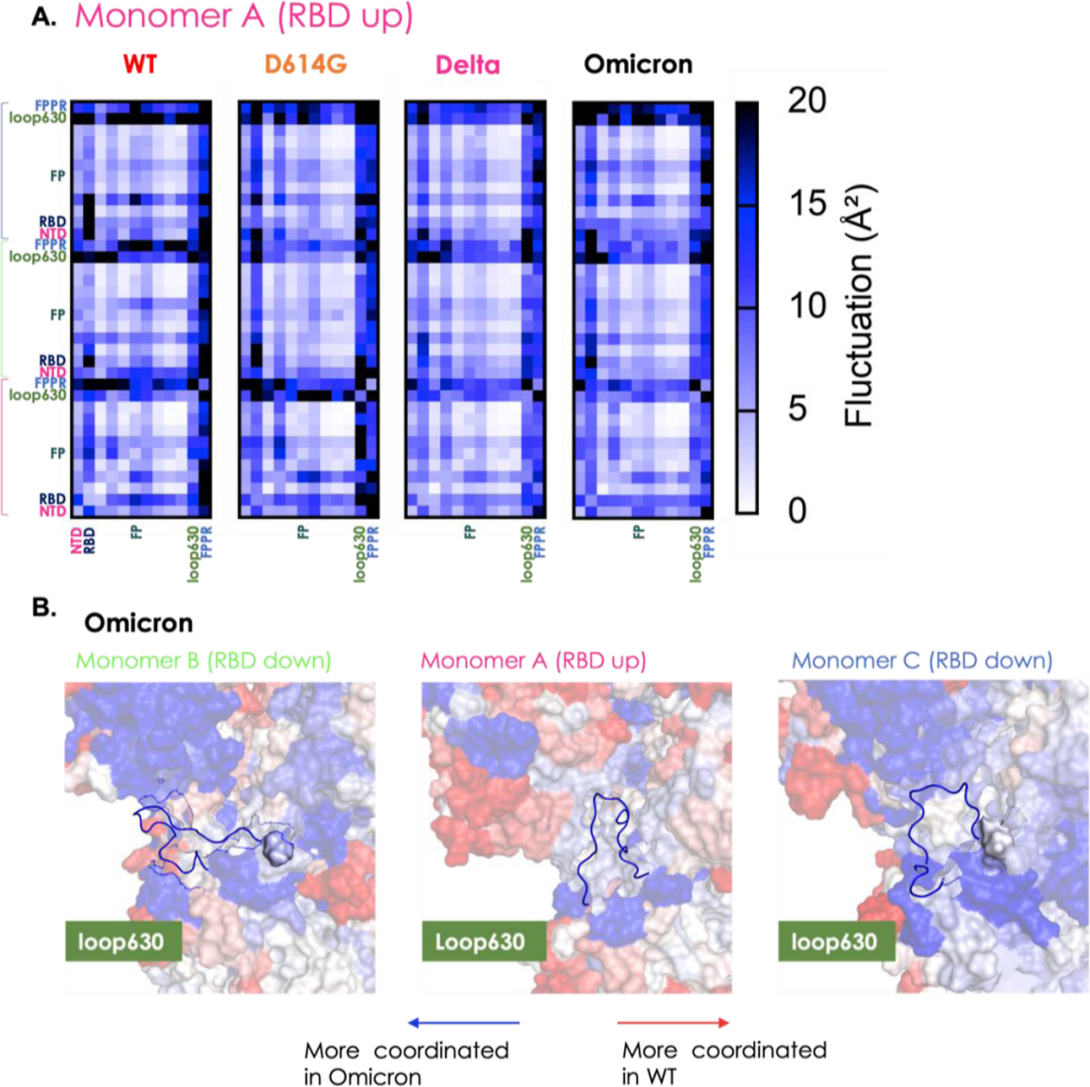
Coordinated motions in terms of distance fluctuations of structural sub-blocks. (A) Simplified block matrix of the coordination matrix in which single blocks report on the overall coordination between structurally defined subdomains. We report here only the DF in blocks for monomer A (RBD up) of WT, D614G, Delta, and Omicron. The matrix is divided considering all domains of the Spike protein (reported in Figure 1). Specifically, NTD, RBD, FP, loop630, and FPPR are highlighted using the color code of Figure 1. (B) Loop630 on the Omicron S-protein: loop630 is a segment which seems to be important for the stabilization of the S-protein in the RBD UP conformation. This substructure was identified in cryo-EM to fold to an ordered structure on passing from the WT to D614G. The Figure reports the point-by-point subtraction of the DF matrix of the Omicron variant from the matrix of the WT, as defined in Figure 3. Considering that almost all of it blue, it can be noted that the loop is certainly much more coordinated in this variant than in original strand, supporting the importance of this loop for the stability of the configuration with one RBD in the UP position (configuration that can be related to infectivity).

We next moved on to analyze the dynamic behavior of the FP and of the region proximal to it, namely FPPR. This site is important for the step following attachment to the cell receptor and to prompt the large conformational changes that eventually lead to the Spike-driven membrane fusion.^27,63^ We used a simplified representation of internal dynamics and coordination patterns, in which the average value of the coordination of all residues within a certain domain with all other substructures is considered. The coordination matrix is thus represented as a simplified block matrix, in which single blocks report on the overall coordination between structurally defined subdomains. Interestingly, both FP and FPPR turn out to increase their dynamic coordination with the rest of the protein upon moving from the WT to all different variants (Figures 6a,b, and S5). Importantly, coordination of these regions is particularly diffuse in the Delta and Omicron variants, indicating that the substructure may be particularly efficient in sensing variations (such as binding to the receptor) in other regions of the protein.

Similar considerations can be applied to loop630, a substructure important for the stabilization of the S-protein in the RBD “up” conformation. This substructure was identified in cryo-EM to fold to an ordered structure on passing from the WT to D614G.^32^

In the variants associated with higher stability, we notice a diffuse coordination for loop 630 in monomers B and C with respect to the variants with lower stability. It is interesting to observe here that the starting structure for this loop is disordered for all variants. Interestingly, Omicron shows a peculiar behavior, in agreement with experimental data:^56^ while the protein is overall more flexible (see above), loop630 is seen to coordinate with the NTD and CTD1 domains within the same protomer. This also reverberates in the larger amount of an ordered secondary structure for loop630 observed for Omicron (Figure 6b).

## Discussion

In this paper, we carried out an extensive analysis of different mutants of the SARS-CoV-2 S-protein. Our aim here was not to thoroughly sample large-scale conformational changes through MD simulations (which, given the number and complexity of the systems under examination, is out of reach) but to shed light on the traits of microscopic dynamics, determined by sequence changes, that can be related to the modulation of motions and differences in the native-state dynamics of the mutants. Importantly, the aforementioned modulations can be revealed even in the absence of major conformational changes. In this context, we note that nanosecond/microsecond time scale residue fluctuations and modulation of protein flexibility have been linked in other cases to the regulation of protein stabilities and activities.^47−51^

Based on our results, we developed a model relating the overall S-proteins’ internal dynamic traits to the sequence modification paths followed by distinct VOCs during their evolution in the first couple of years of diffusion of the virus.

It must be underlined here that the S-protein is an extremely complex system with several mutations sparsely spread throughout the whole structure. In addition, new generations of S-protein are characterized by insertions and deletions, which cause significant variations in the sequence and locally on the structure.

Upon analyzing the data, we initially found that, using traditional metrics correlating local sequence properties with protein structure and dynamics, it was not possible to obtain a general model of protein stability/instability and relate it to the observed traits of diffusion and transmissibility of Spike. In some cases, the same mutation could cause local structural instability in one protein, while resulting in stabilizing in another mutant. In this context, one should furthermore consider that sequence perturbations can have effects that become manifest at distal regions of the protein in an allosteric fashion.

At this point, it is worth underlining that the functional dynamics of a protein (in this case, a certain Spike variant) are a collective property of the molecule.

Based on all these considerations, we reasoned that we could build our work around the hypothesis that ensembles of mutations (not single ones) would exert their effects at a global structural and dynamic level, modulating the ensembles of S-protein that are presented for interaction with the host.

Importantly, we noticed that at the global level (whole protein), the dynamics of the different variants appear to change following the path of somewhat random evolution that characterizes the underlying sequences. In other words, following the time-line of emergence of the various VOCs, one would expect a stepwise modulation of the structural dynamics of their respective S-proteins. However, as noted by Bloom and colleagues (The New York Times “We Study Virus Evolution. Here’s Where We Think the Coronavirus Is Going.” March 28, 2022. https://www.nytimes.com/interactive/2022/03/28/opinion/coronavirus-mutation-future.html), at least initially (2.5 years on the evolutionary scale of a virus can conceivably be considered an early stage), viral evolution selected advantageous sequences through big jumps. Consistent with sequences, the structural dynamics of the S-protein appear to follow this trend. A “random” model for sequence evolution therefore reverberates in the conformational properties of distinct variants that are linked to their ability to spread. Stabilizing mutations may favor diffusion and infectivity by generating versions of the S-protein that are longer-lived (preventing premature S1–S2 dissociation) and more resistant to environmental changing conditions, thus increasing the chances of binding human receptors such as ACE2. At the same time, mutations/insertions/deletions that increase flexibility may also provide an advantage in terms of conformational adaptability of the S-protein to the receptors. Consequently, at the time of writing, Omicron-like variants seem to be prevalent, supporting structural flexibility as one of the factors that decisively favors infectivity. We cannot however rule out that new stabilized variants will emerge in the near future.

In summary, in terms of advantages for the virus to survive the challenges of increasingly trained (by vaccination and acquired immunity) human immune systems, which aim to get rid of the SARS-CoV-2 virus, these random changes could help the virus better escape antibody-mediated first responses, while maintaining (or increasing) its ability to interact with host-cell receptors.^18,64^ Such mechanisms can also provide the virus with an efficient way to scan for sequences with convenient functionally oriented motions. Indeed, a linear, steady-state evolution would be less efficient at exploring the sequence landscape, potentially limiting the capacity to overcome extensive vaccination.

Our dynamics-based results show that the internal coordination of the S-protein can be reconnected to its degree of stability or instability: analyzing pair-DFs, we found specific patterns of extensive residue-pair coordination, particularly pervasive in the whole protein in the variants (D614G, Beta, and Delta) that are experimentally shown to be more stable (these variants elute as single peaks in SDS-page gel electrophoresis).^32,54−56^

In the cases of Omicron, Beta, and Gamma, as well as in the WT, a more globally uncoordinated and generally flexible dynamics is observed, which may be considered as a factor favoring structural instability. In terms of viral evolution and diffusion, both these aspects can be advantageous: increased coordination/stability guarantees persistence of the protein in the active structure in the environment; flexibility, on the other hand, would support a more efficient scan of conformations among which the ones able to recognize and bind ACE2 (and/or other human receptors) can be selected. Extensive flexibility and increased instability could also facilitate the large structural rearrangements of the S-protein required to drive the fusion of the membranes of the host and virus.

In this framework, it is also important to underline that specific functional substructures share the same dynamic traits throughout all variants: these include the motions of the RBD, the FP, and the region preceding it (FPPR), as well as loop630, whose motions stabilize the display of the RBD in the active conformation.^27,32,63^

On the basis of our internal dynamics analyses, here we also developed a ML classification method that allows us to label variants based on a visual representation of their dynamics, automatically reconnecting sequences with biophysical properties.

Overall, we propose a model whereby the jumps in sequence evolution that have characterized the first years of SARS-CoV-2 diffusion are reflected in the variations of the microscopic native dynamics of the encoded S-proteins. In this model, the events of Spike dynamics modification are not sequential and deterministic. A critical feature of our model is that, while we observe a direct coupling between the motions of the RBD, the FP, and the FPPR (hinting to a conserved conformational preorganization of these functionally fundamental substructures), the global dynamics of the rest of the protein appears to rearrange to provide increased stability or increased flexibility.

These two factors can be considered alternative mechanisms to favor S-protein/ACE2 interactions, being at the same time convenient and advantageous.

Our approach may represent a means to characterizing the dynamic properties of different forms of S-protein form distinct VOCs: dynamics are modulated by sequence modifications but retain the traits necessary for the selection of conformational states that favor receptor recognition and binding. The ML model can conveniently intervene in the classification of potentially emerging new variants as STABLE or UNSTABLE, linking this property to the ability of the protein to guide viral-host recognition and infection.

An ideal evolution of this work could be the development of a large database of simulated Spike variants where MD data are integrated with information on their infectivity profiles: in principle, this database should also include mutants that do not improve functions over previous generations. This would proficiently help develop rational predictive models of the effect of future variants and even design possible new antigens for vaccine update. Work in this direction has been carried out using extensive sequence information and ML to predict new evolutionary variants.^65−67^

The generation of long trajectories for a number of variants would certainly permit to develop structure–function relationships for the S-protein: in this case, similar to what is done with small molecules, having examples with different degrees of activity will be decisive to train predictive models. We suggest that methods integrating MD data with sequence analysis (evolution, coevolution, etc.) and AI approaches will have a strong impact in the coming years.

While the production of MD trajectories is certainly still a bottleneck, together with other approaches based on sequence analysis, evolutionary investigations, and the application of different studies of structure–function relationships, this could enrich our knowledge of the physico-chemical determinants of evolution of certain protein forms, relating them to their functions in the context of viral diffusion. While based on the case of SARS-CoV-2 S-protein, our models and considerations are fully general and readily transferable to other targets and contexts.

## Materials and Methods

### Preparation of S-Protein Variants

Fully glycosylated S protein variants simulated in this work were variously derived from simulations described by Grant et al.^68^ based on the Cryo-EM structure of the WT S protein at the Protein Data Bank (PDB) entry 6VSB,^1^ wherein one RBD is in the “up” conformation and the other two are “down”. All mutations, including the “reference” D614G, were introduced using the “mutations wizard” in the PyMOL molecular modeling package (Schrodinger LLC): rotamers of non-glycine side chains were chosen from the first suggested option for S protomer A, and then, where possible, we have sought to adopt the same rotamers for protomers B and C. Histidine tautomers and disulfide bridges were retained as in our reference simulations. In the Alpha variant S protomers, mutant histidines 681 and 1118 were introduced with protonation at Nε2, and mutant aspartate 570 side chains were left unprotonated. Mutant lysine 484 sidechains (B.1.1.28 variant; E484K variant) were left protonated.

Consistent with our reference simulations,^17,18,68^ all three protomers were modeled without gaps, from Ala27 in the NTD to Asp1146 just downstream of heptapeptide repeat 1 (HR1); −NH_3_^+^ and −COO^–^ caps were added, respectively, at N- and C-termini of each protomer.

In the case of the Alpha variant, gaps left by deletions in all three protomers were replaced with artificially long C–N bonds; systems were then allowed to relax with a 400-step preminimization cycle in vacuo (200 steepest descent + 200 conjugate gradient), using the AMBER platform’s *sander* utility (version 18),^64^ in which harmonic positional restraints (*k* = 5.0 kcal mol^–1^ Å^–2^) were applied to all atoms except those in the five residues on either side of the gap. Distortions and clashes introduced with the glycosylated Ser13–Pro26 fragment were resolved using a similar approach.

The same method was used to model deletions of Delta (del157-158) and Omicron (del69-70, del143-145, and del212).

In the case of Omicron, there is also an insertion of three new amino acids (214EPE). This was modeled again using Pymol by inserting the three amino acids into the sequence and then relaxing the system with a 400-step preminimization cycle in vacuo (200 steepest descent + 200 conjugate gradient), using the AMBER platform’s sander utility (version 18),^69^ in which harmonic positional restraints (*k* = 5.0 kcal mol^–1^ Å^–2^) were applied to all atoms except those in the five residues on either side of the insertion.

### MD Simulation Details

After preparation, glycosylated S protein structures were solvated in a cuboidal box of TIP3P water molecules using AMBER’s tleap tool; where necessary, Na^+^ or Cl^–^ ions were added accordingly to neutralize the charge. *N*-Glycosylated asparagines and oligosaccharides were treated using the GLYCAM-06j forcefield,^70^ whereas ions were modeled with parameters by Joung and Cheatham.^71^ To all other (protein) atoms, we applied the *ff14SB* forcefield.^72^ Starting structures and topologies for all simulated variants are electronically provided as Supporting Information.

On each glycosylated S protein variant, we conducted four independently replicated atomistic MD simulations, using the *AMBER* package (version 18): each replica consisted of two 300-step rounds of minimization, 2.069 ns preproduction, and 1 μs production. The *sander* MD engine^69^ was used in the earlier stages of preproduction; thereafter, we switched to the GPU-accelerated *pmemd.cuda*.^69^

### Details on MD Preproduction

Prior to the production stage, every independent MD replica for every S variant goes through a series of preproduction steps, namely minimization, solvent equilibration, system heating, and equilibration. The first two are conducted using the *sander* utility, after which the GPU-accelerated *pmemd.cuda* is invoked instead.

Minimization takes place in two 300-step rounds, the first 10 of which use the steepest descent algorithm and the last 290 conjugate gradient. In the first round, we only minimize the backbone Hα and H1 hydrogens on amino acids and monosaccharides, respectively, restraining all other atoms harmonically (*k* = 5.0 kcal mol^–1^ Å^–2^). Thereafter, all atoms are released, including solvent and ions.

Solvent equilibration occurs over 9 ps with a time step of 1 fs; the ensemble is *NVT*, with temperatures in this case enforced by the Berendsen thermostat.^73^ Positions of non-solvent atoms are harmonically restrained (*k* = 10 kcal mol^–1^ Å^–2^). Solvent molecules are assigned initial random velocities to match a temperature of 25 K. Fast heating to 400 K (coupling: 0.2 ps) is performed over the first 3 ps; the solvent is then retained at 400 K for another 3 ps and cooled back down to 25 K over the last 3 ps, more slowly (coupling: 2.0). The cutoff for determining Lennard-Jones and Coulomb interactions remains at 8.0 Å for this and all subsequent stages, as does the particle mesh Ewald method^74^ to determine Coulomb interactions beyond this cutoff. SHAKE constraints^70^ are not applied at this stage but are always present thereafter.

For system heating, the time step is increased to 2 fs and, while continuing in the *NVT* ensemble, temperatures are now enforced by the Langevin thermostat^71^ (which remains in place for all subsequent stages). With an initial collision frequency of 0.75 ps^–1^, the system is heated from 25 to 300 K over 20 ps: all atoms are free to move except amino acids’ Cα atoms, which are positionally restrained with *k* = 5 kcal mol^–1^ Å^–2^.

For equilibration, the ensemble is switched to *NpT* (*p* = 1 atm; Berendsen barostat coupling: 1 ps), and the system is simulated for a further 2040 ps. The thermostat’s collision frequency is kept lower than in the production stage (1 ps^–1^). Restraints on Cα atoms are lifted gradually: *k* = 3.75 kcal mol^–1^ Å^–2^ for the first 20 ps; 1.75 kcal mol^–1^ Å^–2^ for the following 20 ps; none thereafter.

### Details on MD Production

The 1 μs production stage is carried out in the *NpT* ensemble (*T* = 300 K; *p* = 1 atm) using a 2 fs time step; a cutoff of 8.0 Å is applied for the calculation of Lennard-Jones and Coulomb interactions alike. Coulomb interactions beyond this limit are computed using the particle mesh Ewald method.^68^ All bonds containing hydrogen are restrained using the SHAKE algorithm.^75^ Constant pressure is enforced via the Berendsen barostat^76^ with a 1 ps relaxation time, whereas temperature is stabilized by the Langevin thermostat^73^ with a 5 ps^–1^ collision frequency.

### Residue-Pair DFs

To understand the impact mutations on the internal dynamics of SARS-CoV-2, we conducted the DF analysis.

To compute the matrix of DFs, we used the 4 μs metatrajectory available for each studied system, obtained by concatenating the MD replicas of each specific protein; in this framework, each element of the matrix corresponds to the DF parameters
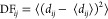
1where *d*_*ij*_ is the time-dependent distance of the Cα atoms of amino acids *i* and *j* and the brackets indicate the time-average over the trajectory. The advantage of this parameter is its invariant nature under translations and rotations of the molecules and, different from the covariance matrix, that it does not depend on the choice of a particular protein reference structure.

DF was calculated for every pair of residues during the trajectory. This parameter characterizes residues that move in a coordinated fashion, and it is actually able to reflect the presence of specific coordination patterns and quasi-rigid domains motion in the protein of interest. In particular, pairs of amino acids belonging to the same quasi-rigid domain or highly coordinated at a distance are associated with small DFs and vice versa.

### DFs in Blocks

To further analyze the coordination patterns among distinct subdomains of the protein, we first subdivided the structure into domains, also called blocks in our definition, according to the annotation reported in Figures 6a and S5. Here, we evaluate the degree of interdomain coordination among different blocks and the contribution of each single block to the overall internal dynamics of the protein. DF for each domain (block) is calculated from the full DF matrices reported above. The latter is in fact simplified by combining the contributions of residues assigned to a certain domain (block) based on the sequence definition from Figures 6a and S5. The cumulative DF value associated with each block is then obtained by averaging all terms for each residue grouped in the block.

### Difference between DF Matrices

To further compare fluctuation matrices, we calculated the difference matrix, obtainable by subtracting the matrix for one particular protein from the DF matrix of the Wuhan (WT) molecule, used as a reference for all such calculations (Figure S2). To account for sequence differences due to deletions and insertions, we simply considered the DF matrices of all common structures among the proteins to obtain matrices of the same dimensions. The values of the various difference matrices, reporting on how the internal dynamics of a variant changes with respect to the WT, are then summed by column: the obtained parameter reports on the increased or decreased global coordination of the residue corresponding to that column, with respect to the WT. The parameter is then projected with using the color code reported in Figure 3 on the 3D structure.

### RBD Fluctuations

To follow the fluctuations of the RBD during the simulation, we focused the sampling along a two-dimensional progress coordinate: (1) the difference in the center of mass of the spike core to the RBD (distances parameter) and (2) the angle defined by these two regions of the S-protein (angles).

### Distances

We used the CPPTRAJ and the command distances (https://amberhub.chpc.utah.edu/distance/) to calculate the distances between the center of mass of atoms in “mask1” to atoms in “mask2”. The atoms in “mask1” are the atoms of the RBD, and the “mask2” includes residues of the core of the Spike (849–881 and 945–1045 of each protomer of the protein).

### Angles

To construct the angle between these two masks, we first used the vector command of CPPTRAJ (https://amberhub.chpc.utah.edu/vector/) to keep track of a vector value (and its origin) of each mask over the trajectory and afterward, we perform the vector product (https://amberhub.chpc.utah.edu/vectormath/) to get the angles between the two previously calculated vectors (using the option “dotangle” to calculate the angle from dot-product between the two vectors; vectors will be normalized).

### CNN-ML

#### Preparing DF images

The trajectories from the MD simulations were directly submitted to the DF matrix calculation using the above-reported procedure. Specifically, we extracted the DF every 10 ns, starting from the very first not equilibrated ones till the last of the dynamics. We ended up with a total number of 2816 DF matrices. We then used an in-house developed *Gnuplot* script (available in the Supporting Information) to prepare the images with a dimension of 300 × 300 pixels using a white-blue-black color palette. Colors tending toward white indicate a DF of ∼0 Å^2^, while black ones indicate a DF of ∼10 Å^2^. The halfway point (i.e., DF of ∼5 Å^2^) is represented with blue nuances.

#### Preparing the CNN Model

Image recognition through CNN was elaborated using a modified version of the readily available VGG19 model since it demonstrated to be one of the best compromises between computational cost and accuracy and can be directly imported in Python using TensorFlow (TF) (the Python script is available in the Supporting Information).^62^

The architecture of the VGG19 model was maintained unaltered, while we modified the dimensions of layers in order to accommodate the 300 × 300 pixels of the input DF image. Furthermore, the imported images were again rescaled to the dimension of the VGG19 layers and normalized according to the standard pixel values which can range from 0 to 255. This step aims to exclude possible scaling errors introduced during the *Gnuplot* image preparation from the numerical matrix.

We set the classification mode to “binary” (class_mode) and the number of samples propagated through the network was set to 32 (batch_size). To provide a measure for the goodness of the method, we used *ImageNet* (for ImageNet, see: https://image-net.org/about.php) weights, widely recognized to be the standard for image classification problems.

The last layer of our VGG19-modified model provides the prediction output using a single layer on which the “sigmoid” activation function σ(*z*) acts
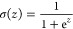
2

We selected this function since it is the standard for binary classifications: given its existence only between 0 and 1, it constitutes the natural choice for binary problems. We compiled the model by using a “binary cross-entropy” loss function and using the “Adam” algorithm for the stochastic optimization.^77^ Last, in order to avoid model overfitting, we introduced an early stopping monitor which stops training the model if the validation loss starts increasing during five consecutive epochs. However, we never experienced strong increases in propagation of the loss function to justify an intervention of the monitor. Moreover, the specific placement of the five max pooling layers reduced the computation time and memory usage, by also limitating the probability to get into overfitting issues.

#### Training of the Model and Test with Internal Data

To train the model, we selected the DF images coming from the last equilibrated 200 ns of each replica, for a total number of 672 images, which were manually divided between test (20%), train (64%), and validation (16%) sets. Within these sets, we operated a manual classification in order to define the two main classes of interest in our model: STABLE variants (Beta, D614G, Delta, and Delta^+^) and UNSTABLE variants (WT, Omicron, Alpha, and Gamma). We trained our model for 20 epochs using the data sets, and we obtained complete training and validation in 53 s, with an average of 153 ms/step. This result is extremely promising and is mainly due to the TF parallelization of using GPU.

We next tested the just trained model with data arriving from the same equilibrated portion of dynamics but not used during the train and validation steps. We got 100% accuracy, taking only 3 s to scan all 134 DF test images. This result was also checked through the classification report and confusion matrix analysis, in order to validate the goodness of the predictions.

#### Test of Model with External Data

We submitted to the trained model a new data set prepared by taking 20 unseen DF matrix images from MD replicas of the variants involved in this study and never used for the previous training of the model. We selected 10 images coming from the STABLE variants and 10 from the UNSTABLE ones. The manual choice we operated was specifically directed in order to choose within the first non-equilibrated parts of each trajectory. The aim was to prove that—once the model is trained—our method can be extended to other new variants without the need to use long MD simulations. Those images were first submitted to the same scaling and normalization steps as performed for the other sets of data (see above). The only difference we introduced was in the number of samples propagated through the network, which was set to 1 (batch_size) since we needed to predict each submitted image. Moreover, in accordance with the just printed classification report, we were able to assign the UNSTABLE class if the prediction assumed values above 0.5, while if below, the STABLE class was inferred.

The test on external data was extremely fast and only took 3 s to complete all 20 classifications, with a final accuracy of 100% for each of them.

## Supporting Information Available

All matrices of DFs of all variants, point-by-point subtraction of the DF matrix of each variant, neural-network architecture and the summary of the model with the confusion matrix and Cohen’s kappa coefficient, all protein DFs of all variants in blocks, and AMBER input files, topologies, and restart (rst) files for each variant are provided as a GDRIVE directory at the link: https://drive.google.com/drive/folders/1yvBEx_N3rhCOwoE-LVX01puSXZ16SAfm?usp=share_link. The drive folder is further divided into two folders: “MD” which contains the material for each simulation which is divided into folders for each variant containing the starting solvated topology file (Spike-$(variant).solv.top), the production script (prod.in), and the equilibration restart (equil3.rst) and “CNN-ML” which contains the scripts required for ML. Given their large dimensions, full trajectories are available upon request (PDF)
