# Supplementary material for: Conformational Behavior of SARS-Cov-2 Spike Protein Variants: Evolutionary Jumps in Sequence Reverberate in Structural Dynamic Differences
Source: J Chem Theory Comput. 2023 Mar 16;19(7):2120–34. doi: 10.1021/acs.jctc.3c00077 (PMC10029694; doi:10.1021/acs.jctc.3c00077)
Supplement: Supplementary file 1 — ct3c00077_si_001.pdf [file ct3c00077_si_001.pdf]

Supporting Information

The Conformational Behaviour Of SARS-Cov-2 Spike Protein Variants: Evolutionary Jumps In Sequence Reverberate In Structural Dynamic Differences

*Alice Triveri,[1] Emanuele Casali,[1] Elena Frasnetti,[1] Filippo Doria,[1] Francesco Frigerio,[2] Fabrizio Cinquini,[3] Silvia Pavoni,[2] Filippo Marchetti,[1] Stefano A. Serapian,[1] Giorgio Colombo[1],**

1) Dipartimento di Chimica, Università degli Studi di Pavia, via Taramelli 12, 27100 Pavia (Italy)

e-mail: g.colombo@unipv.it

2) Department of Physical Chemistry, R&D Eni SpA, via Maritano 27, 20097 San Donato Milanese (Mi), Italy

3) Upstream & Technical Services – TECS/STES – Eni Spa, via Emilia 1, 20097 San Donato Milanese (Mi), Italy

TABLE OF CONTENTS

1. Distance fluctuations of all the variants Pag. S2
2. Point-by-point subtraction of the DF matrix of each variant Pag. S3
3. Neural-Network architecture Pag. S4
4. Summary of the model Pag. S5
5. Confusion matrix and Cohen's kappa coefficient Pag. S6
6. Angles and Distance Analysis of RBD Pag. S7
7. Block-Based Distance Fluctuations of all the variants Pag. S8

1. **Distance fluctuations of all the variants.**

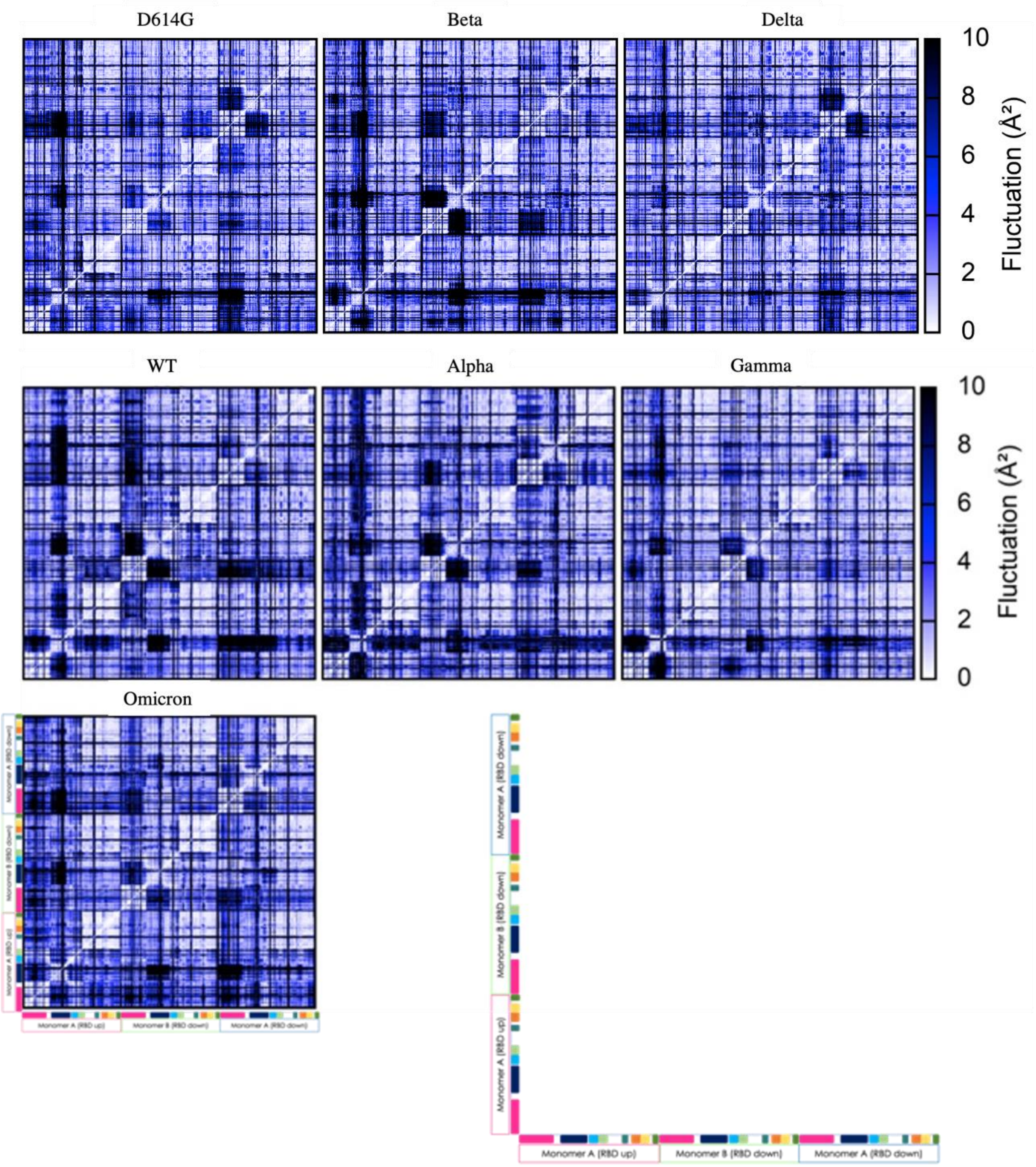

Figure S1. Distance fluctuation and the zoom of the domains.

2. Point-by-point subtraction of the DF matrix of each variant

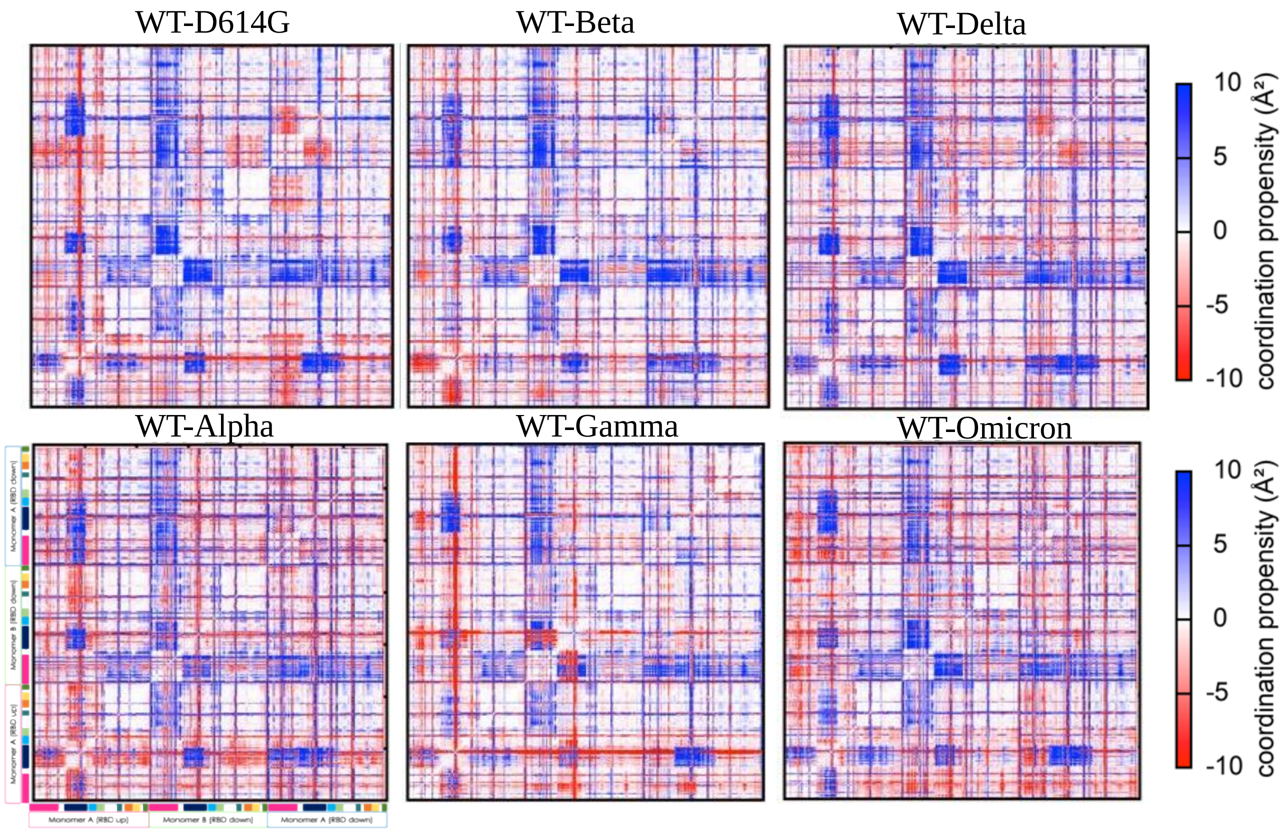

Figure S2. Point-by-point subtraction of the DF matrix of each variant from the WT variant.

3. Neural-Network architecture

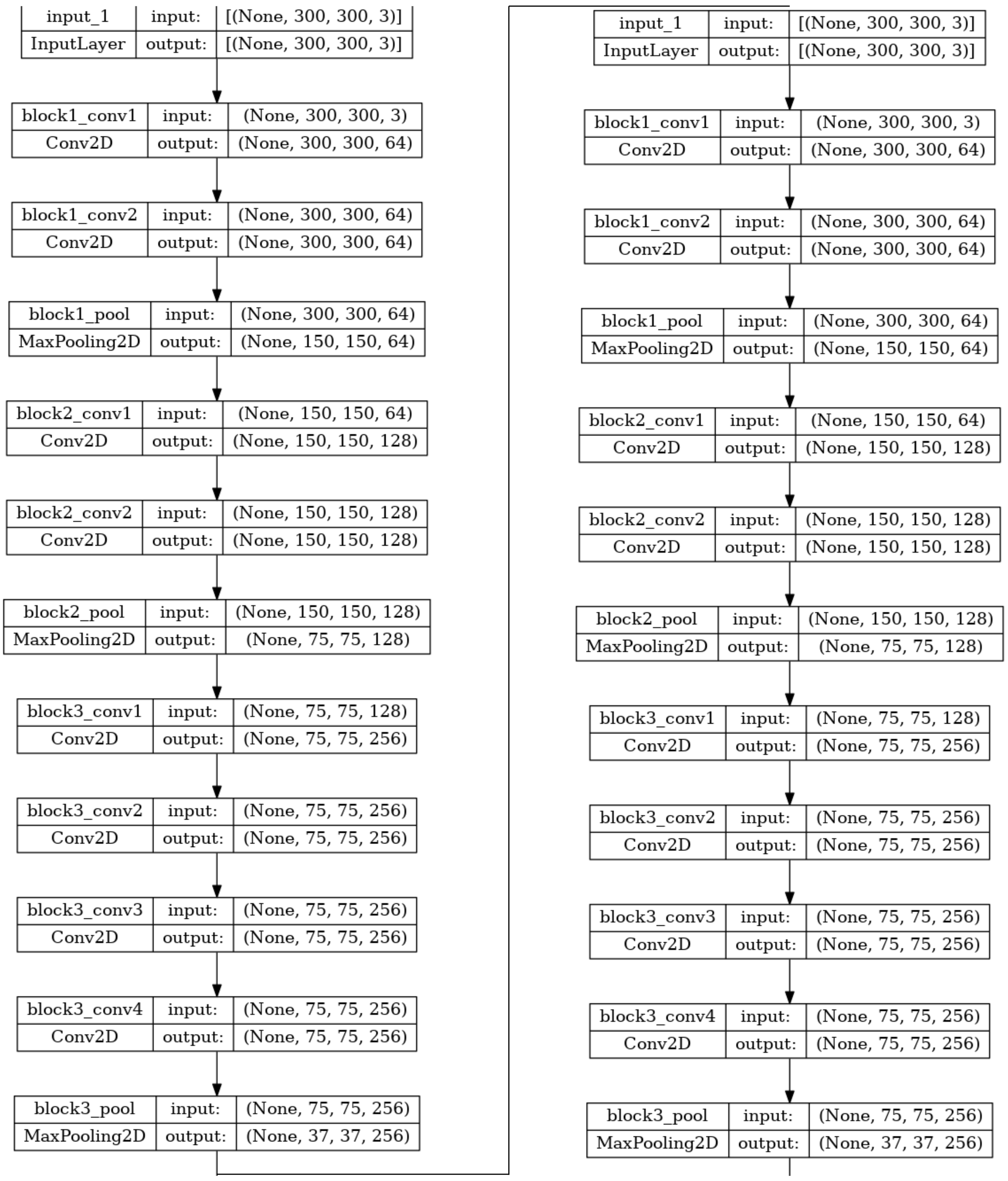

Figure S3. Plot of the modified VGG19 Neural Network Model Graph

4. Summary of the model

```
Layer (type)                 Output Shape              Param #
=================================================================
input_1 (InputLayer)         [(None, 300, 300, 3)]     0

block1_conv1 (Conv2D)        (None, 300, 300, 64)      1792

block1_conv2 (Conv2D)        (None, 300, 300, 64)      36928

block1_pool (MaxPooling2D)   (None, 150, 150, 64)      0

block2_conv1 (Conv2D)        (None, 150, 150, 128)     73856

block2_conv2 (Conv2D)        (None, 150, 150, 128)     147584

block2_pool (MaxPooling2D)   (None, 75, 75, 128)       0

block3_conv1 (Conv2D)        (None, 75, 75, 256)       295168

block3_conv2 (Conv2D)        (None, 75, 75, 256)       590080

block3_conv3 (Conv2D)        (None, 75, 75, 256)       590080

block3_conv4 (Conv2D)        (None, 75, 75, 256)       590080

block3_pool (MaxPooling2D)   (None, 37, 37, 256)       0

block4_conv1 (Conv2D)        (None, 37, 37, 512)       1180160

block4_conv2 (Conv2D)        (None, 37, 37, 512)       2359808

block4_conv3 (Conv2D)        (None, 37, 37, 512)       2359808

block4_conv4 (Conv2D)        (None, 37, 37, 512)       2359808

block4_pool (MaxPooling2D)   (None, 18, 18, 512)       0

block5_conv1 (Conv2D)        (None, 18, 18, 512)       2359808

block5_conv2 (Conv2D)        (None, 18, 18, 512)       2359808

block5_conv3 (Conv2D)        (None, 18, 18, 512)       2359808

block5_conv4 (Conv2D)        (None, 18, 18, 512)       2359808

block5_pool (MaxPooling2D)   (None, 9, 9, 512)         0

flatten (Flatten)            (None, 41472)             0

dense (Dense)                (None, 1)                 41473

=================================================================
```

The model summary is a textual way to report information about the layers and their order in a convolutional network model. It also provides the output shape of each layer and the number of parameters propagated through the network. In our case, we can observe that an increasing number of parameters is obtained during the convolutional stage as a consequence of mathematical convolution in each layer. Conversely, max pooling layers do not increase the number of parameters, being involved only in reducing the computational cost and controlling overfitting. Finally, the number of parameters is brought to conversion through the fully-connected fatten layer and is kept unchanged in the dense one, where the sigmoid function acts to provide the prediction.

5. Confusion matrix and Cohen’s kappa coefficient

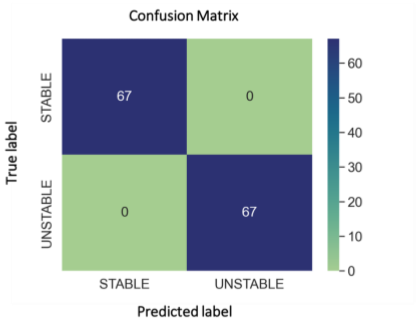

$$\kappa = \frac{Pr(a) - Pr(e)}{1 - Pr(e)}$$

$$Pr(a) = \frac{TP + TN}{T}$$

$$Pr(e) = \frac{(TP \cdot FP \cdot FN) + (TN \cdot FP \cdot FN)}{T^2}$$

Figure S4. Confusion matrix and Cohen’s kappa expression

The confusion matrix is widely used in artificial intelligence and machine learning approaches. It returns a representation of statistical classification accuracy. Each column of the matrix represents the predicted values, while each row represents the actual true values. In our case we can see a perfect match between predicted stable cases and true one, as well as those belonging to the other subset. The Cohen's kappa coefficient, whose expression is reported in the equation above, is a statistical coefficient representing the degree of concordance in accuracy and reliability of a statistical classification. In our case κ is equal to 1, which means that the statistic represents the optimal case. (TP = true positive, TN = true negative, FP = false positive, FN = false negative, T = total)

6. Angles and Distance Analysis of RBD

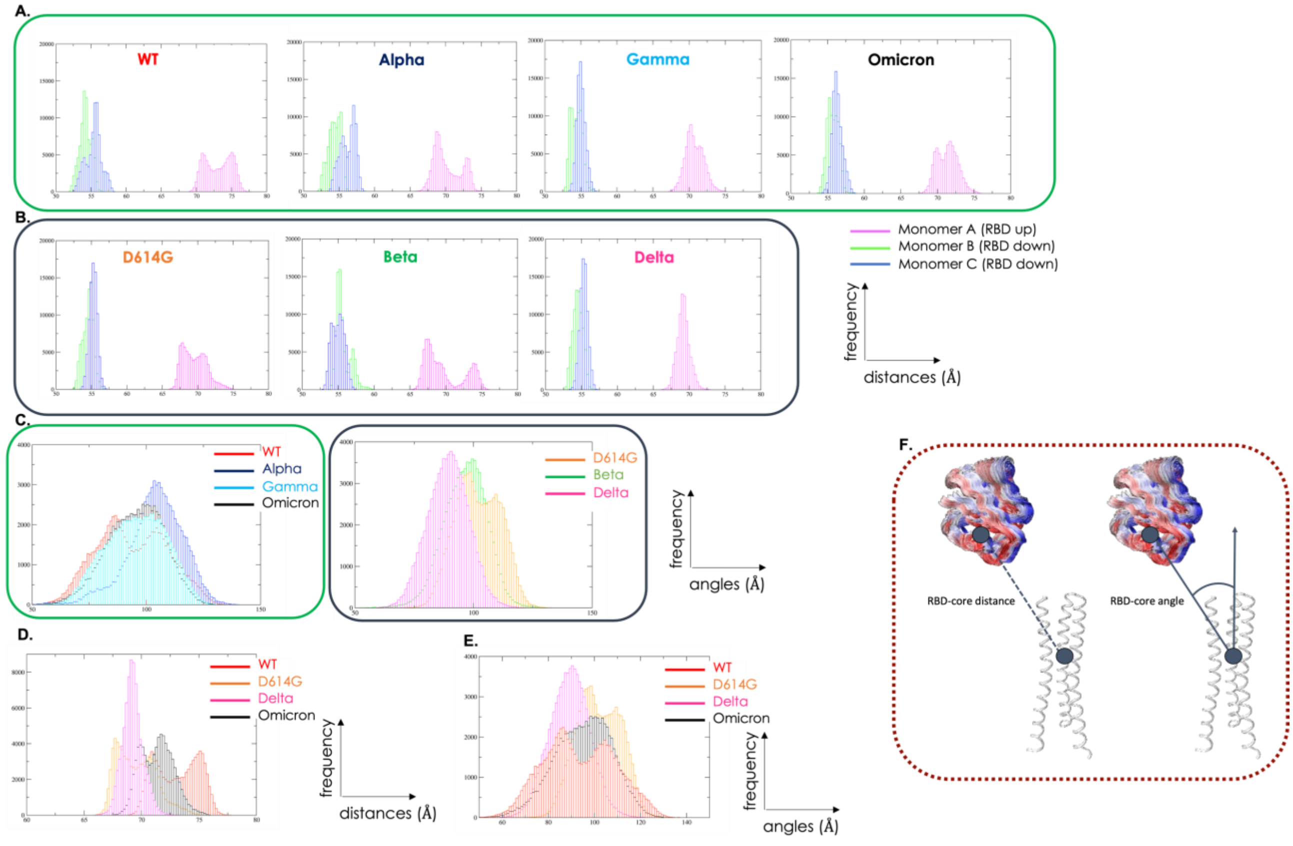

The Dynamics of RBD: comparison of Distance and Angles analysis.
In green hues the UNSTABLE variants and in grey hues the STABLE variants. (A) Distance between the centers of mass (COMs) of the spike core and of the RBD of the UNSTABLE variants (WT, Alpha, Gamma and Omicron) represented in histograms. In magenta the fluctuation of monomer A with the RBD in the UP position, in green the monomer B (RBD down) and in blue the monomer C (RBD down). (B) Distance between the centers of mass (COMs) of the spike core and of the RBD of the STABLE variants (D614G, Beta and Delta). The graphs report on the x-axis the distances (Å) and on the y-axis the frequencies.
(C) The 3D angle between the spike core and of the RBD up in histograms. In the green box the UNSTABLE variants and in grey the STABLE variants. Histograms have on the x-axis the angles (Å) and on the y-axis the frequencies. In (D) and (E) are reported the comparisons between the distances (D) and the angles (E) in the most representative variants (WT, D614G, Delta and Omicron). The inset reports a simplified cartoon representation of the variables mentioned.

7. Distance fluctuations of all the variants in blocks

These Distance fluctuations matrices can be further simplified by dividing the protein according to its domains (the so-called "BLOCKS" that represent the domain of the spike protein for each monomer). Here we report these blocks:

1. NTD
2. RBD
3. CTD1
4. CTD2
5. Residues between the CTD2 and the FP
6. FP
7. Residues between the FP/FPPR and the HR1
8. HR1
9. CH
10. CD
11. Loop630
12. FPPR

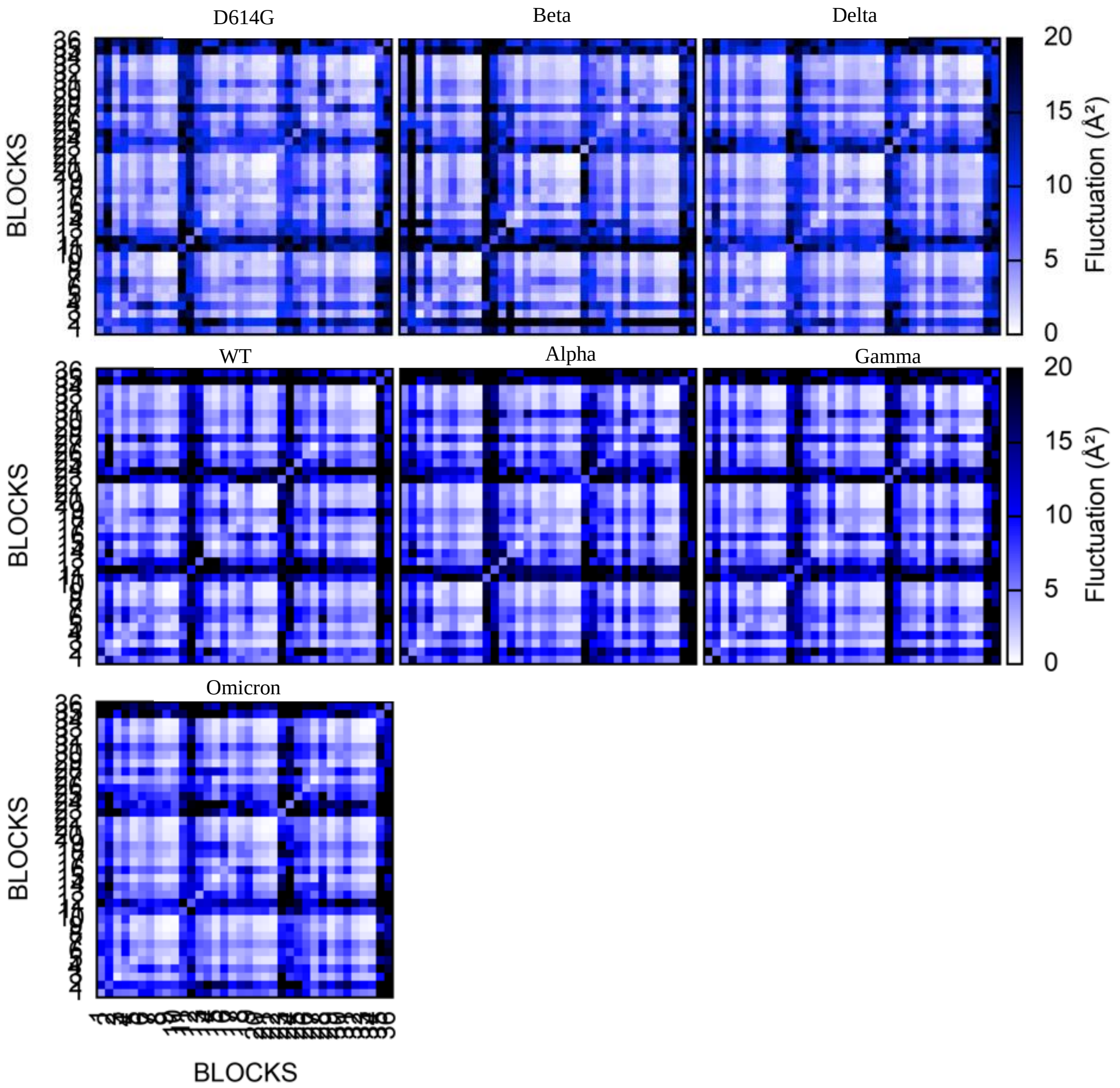

Figure S5. Distance fluctuations of all the variants divided in blocks correspond to the domains of the protein.
